# Colorectal Cancer Screening Prevalence and Adherence for the Cancer Prevention Project of Philadelphia (CAP3) Participants Who Self-Identify as Black

**DOI:** 10.3389/fonc.2021.690718

**Published:** 2021-07-30

**Authors:** Elizabeth L. Blackman, Camille Ragin, Resa M. Jones

**Affiliations:** ^1^Department of Epidemiology and Biostatistics, College of Public Health, Temple University, Philadelphia, PA, United States; ^2^Cancer Prevention and Control Program, Fox Chase Cancer Center- Temple University Health System, Philadelphia, PA, United States; ^3^African Caribbean Cancer Consortium, Philadelphia, PA, United States

**Keywords:** screening, colon, disparities (health racial), immigrant health, colorectal cancer, African American, cancer, cancer prevention

## Abstract

**Introduction:**

Colorectal cancer is the third leading cause of cancer-related deaths among Black men and women. While colorectal cancer screening (CRCS) reduces mortality, research assessing within race CRCS differences is lacking. This study assessed CRCS prevalence and adherence to national screening recommendations and the association of region of birth with CRCS adherence, within a diverse Black population.

**Methods:**

Data from age-eligible adults, 50–75 years, (N = 357) participating in an ongoing, cross-sectional study, was used to measure CRCS prevalence and adherence and region of birth (e.g., Caribbean-, African-, US-born). Prevalence and adherence were based on contemporaneous US Preventive Services Task Force guidelines. Descriptive statistics were calculated and adjusted prevalence and adherence proportions were calculated by region of birth. Adjusted logistic regression models were performed to assess the association between region of birth and overall CRCS and modality-specific adherence.

**Results:**

Respondents were 69.5% female, 43.3% married/living with partner, and 38.4% had <$25,000 annual income. Overall, 78.2% reported past CRCS; however, stool test had the lowest prevalence overall (34.6%). Caribbean (95.0%) and African immigrants (90.2%) had higher prevalence of overall CRCS compared to US-born Blacks (59.2%) (p-value <0.001). African immigrants were five times more likely to be adherent to overall CRCS compared to US-born Blacks (OR = 5.25, 95% CI 1.34–20.6). Immigrants had higher odds of being adherent to colonoscopy (Caribbean OR = 6.84, 95% CI 1.49–31.5; African OR = 7.14, 95% CI 1.27–40.3) compared to US-born Blacks.

**Conclusions:**

While Caribbean and African immigrants have higher prevalence and adherence of CRCS when compared US-born Blacks, CRCS is still sub-optimal in the Black population. Efforts to increase CRCS, specifically stool testing, within the Black population are warranted, with targeted interventions geared towards US-born Blacks.

## Introduction

The American Cancer Society (ACS) estimates there will be about 147,950 new colorectal cancer (CRC) cases diagnosed in the US in 2020 and about 53,200 CRC deaths (1, 2). CRC is the third most frequently diagnosed cancer among Black men and women as well as the third-leading cause of cancer-related deaths (3). Further, racial disparities exist, with Non-Hispanic Blacks having the highest CRC incidence and mortality rates, when compared to other racial groups (3). Importantly, Blacks are a heterogeneous racial group and 10% of the US Black population are immigrants from the Caribbean and Africa (3, 4). Further, second generation immigrants make up an additional 8% of the population, subsequently making approximately 20% of Black population, immigrant-blacks and their children (5). Previous work has shown explicit differences in CRC mortality within the heterogeneous Black population in the US (6–8).

CRC is one of few cancers where mortality can be reduced 9–32% (9–14) with regular screening (1, 3, 15, 16). The U.S. Preventive Services Task Force (USPSTF) (16) and the ACS (15) have set guidelines for CRC screening (CRCS) for average-risk adults, ages 45–75, to ultimately reduce mortality. These recommendations include having a stool test within the last year, flexible sigmoidoscopy or computed tomography (CT) colonography in the last 5 years, or a colonoscopy within the last 10 years. While the importance of CRCS has been noted in the published literature, adherence to US CRCS guidelines (16) is not ideal and should be improved (17). While CRCS adherence appears similar between Whites and Blacks, the published literature provides evidence of an ethnic/racial disparity (18–27). Levels of adherence to any modality of CRCS (24–26), stool test [fecal occult blood test (FOBT) or fecal immunological test (FIT)] (18–20, 22, 23) and colonoscopy (19–23, 27), have been consistently higher in whites when compared to any other racial ethnic group.

Research assessing within race differences for CRCS prevalence and adherence is lacking. Therefore, using contemporaneous USPSTF guidelines at the inception of this research, this study determines the prevalence of CRCS and adherence to national screening recommendations among a heterogeneous population of Blacks, aged 50–75 years, participating in the Cancer Prevention Project of Philadelphia (CAP3). In addition, the association of region of birth (i.e., US, Caribbean or African born) and CRCS adherence was also assessed.

## Methods

This study used a subset of data from the ongoing CAP3 study for individuals recruited from September 2012 to August 2019. Methods for CAP3 have been described previously (28). Briefly, CAP3 recruited individuals in the Philadelphia metropolitan area, where the Black community is the largest minority group (~44% of the total population) (29) consisting of US-born, Caribbean-born, and African-born Blacks. This study was reviewed and approved by the Institutional Review Board at Fox Chase Cancer Center. All participants provided informed consent.

For the parent study, enrollment was limited to English speakers age 18+, who do not have a cancer diagnosis at the time of study enrollment. Having other comorbidities (i.e. hypertension, diabetes, etcetera), did not prevent study participation. For the current study, only individuals who were age eligible for CRCS (i.e., 50–75 years, the USPSTF recommendation in 2012 when the study was initiated) and responded to CRCS questions were included in the analysis (N = 357).

### Data Collection

Questionnaires were administered *via* in-person interviews by trained research staff.

#### Measures

CRCS questions were adapted from the 2011 Behavioral Risk Factor Surveillance System (BRFSS) and the National Health and Nutrition Examination Survey (NHANES), both developed by the CDC (30, 31). Five questions in the CAP3 questionnaire provide data on CRCS-related prevalence and adherence. Specifically, participants were asked if they ever had a stool-based test and an endoscopic method of CRCS. If the individuals had received an endoscopic procedure, they were asked to specify whether it was flexible sigmoidoscopy or colonoscopy. The timing of each CRCS modality was also asked with the following response categories: within the past year (anytime less than 12 months ago); within the past two years (1 year but less than 2 years ago); within the past 3 years (2 years but less than 3 years ago); within the past 5 years (3 years but less than 5 years ago); within the past 10 years (5 years but less than 10 years ago); 10 or more years ago; don’t know/not sure, and refused (31).

Country of birth, a single, open-ended question was asked of all participants. This variable was then categorized into a 3-level variable to describe ethnicity.

Demographic variables included: age, sex, marital status, education, income, and ethnicity. Other variables included: healthcare coverage, whether the respondent had health insurance; primary care provider status, whether the participant had someone they considered a primary care doctor; and routine physical (whether the participant had a routine physical in the last year). Length of time in the US represents the number of years each respondent has lived in the US.

### Coding

#### Primary Outcome Variables

CRCS questions assessing stool test and two endoscopic modalities (i.e., colonoscopy or flexible sigmoidoscopy) were coded dichotomously as “yes” or “no” to reflect screening prevalence—whether people had ever had a stool test, colonoscopy, or any CRCS. A subsequent question was asked to determine time frame from last modality of CRCS, which were used to dichotomously code adherence variables as “never screened/overdue” or “adherent” based on the 2012 USPSTF guidelines (15, 16): stool test (in last year), colonoscopy (in last 10 years), and overall CRCS (stool test in last year, colonoscopy in last 10 years, or flexible sigmoidoscopy in last five years). Flexible sigmoidoscopy prevalence and adherence were not explicitly assessed as it is rarely recommended in clinical practice (15) and very few people reported having the test (n = 9). However, the data for flexible sigmoidoscopy was included in the overall CRCS prevalence and adherence variables.

#### Independent Variable

Country of birth was recoded into a 3-level “region of birth” variable representing “US-born”, “Caribbean-born”, and “African-born”. US-born included individuals that were born in the continental US and US territories around the world; Caribbean-Immigrants included individuals born in Barbados, Grenada, Guyana, Haiti, Jamaica, St. Lucia, and Trinidad and Tobago; lastly, African-Immigrants included individuals born in The Democratic Republic of the Congo, Liberia, Nigeria, Sierra Leone, Togo, and Uganda.

#### Sociodemographic Variables

The following categorical sociodemographic variables were included in analyses: age (“50–64” or “65+” years), sex (“male” or “female”), marital status (“married or member of an unmarried couple,” “divorced, widowed or separated” or “never married”), annual household income (“less than $10,000 to 24,999,” “$25,000 to 49,999,” “$50,000 to 74,999,” “$75,000+,” and “don’t know/not sure”), highest level of education (“<high school,” “high school graduate” “some college,” “ college and beyond,” and “don’t know/refused”), healthcare coverage (“yes” or “no”), having a primary care doctor (“yes” or “no”) and having a routine physical (“within the last year” or greater than a year ago”). For regression analyses the following variables were recoded to have dichotomous responses: marital status (“married or member of an unmarried couple” or “divorced, widowed or separated, never married”), annual household income (“≤$50,000” or “>$50,000”), and highest level of education (“≤high school,” or “>high school”). Length of time in the US, a continuous variable coded to represent years living in the US was also included in the analysis. For US-born Blacks this variable was coded as their age and for immigrants it was coded as length of time they have resided in the US.

### Statistical Analysis

STATA version 13.1 was used to perform all statistical analyses. Descriptive statistics were calculated for the overall population of Blacks as well as stratified by self-reported region of birth. Fisher’s exact or χ^2^ tests were used to assess differences within the Black population; Fisher’s exact test was used when respondent frequency was less than 5. Adjusted proportions for CRCS prevalence and adherence were calculated. Adjustment was done as appropriate based on established confounders in the literature (32–38) and the 10% change-in-estimate criterion (39). Specifically, marital status, level of education, income, healthcare coverage status, primary care provider (PCP) status, and having a routine physical within the last year were confounders and age, sex, and length of time in the US were included as covariates.

Adjusted logistic regression models were run for overall CRCS adherence and modality-specific CRCS adherence. Model specific adjustment was done as appropriate; methods previously described were used to determine covariates and potential confounders to be added to each modality-specific model. All odds ratios were deemed significant given the 95% confidence interval and α = 0.05. Approximately 100 people were needed to detect a significant difference for overall CRCS and colonoscopy adherence at 80% power, with a two-tailed test with α = 0.05.

## Results

Descriptive respondent characteristics are reported in **Table 1**. Overall, respondents were 69.5% female, 43.3% married or a member of an unmarried couple, and 38.4% made less than $25,000 a year and 81.2% had at least a high school diploma. Mean age for the entire study population is 59.7 years (standard error (SE) ± 0.37). Mean length of time in the US for Caribbean immigrants was 24.5 years (SE ±1.3) and 16.7 years for African immigrants (SE ±1.63). African and Caribbean immigrants were less likely to have health insurance when compared to US-born Blacks (63.0, 80.6 and 92.8%, respectively; p <0.001). This trend continued for having a primary care physician (78.3, 80.6, and 87.3%, respectively; p <0.001).

**Table 1:** Respondent characteristics (N = 357).

	Total*	US-Born	Caribbean-Born	African-Born	p-value
	N = 357	n = 208	n = 103	n = 46	
	N (%)	N (%)	N (%)	N (%)	
**Age**					0.468
50–64	269 (75.4)	155 (74.5)	76 (73.8)	38 (82.6)	
65+	88 (24.6)	53 (25.5)	27 (26.2)	8 (17.4)	
**Sex**					0.385
Male	109 (30.5)	68 (32.7)	26 (25.2)	15 (32.6)	
Female	248 (69.5)	140 (67.3)	77 (74.8)	31 (67.4)	
**Marital Status**					
Married or Member of Unmarried Couple	154 (43.3)	62 (29.9)	61 (60.0)	31 (67.4)	**<0.001**
Divorced, Widowed or Separated	119 (33.7)	78 (37.7)	28 (28.0)	13 (28.3)	0.171
Never Married	81 (23.0)	67 (32.4)	12 (12.0)	2 (4.3)	**<0.001**
**Annual Household Income**					
>$10,000–24,999	136 (38.4)	86 (41.7)	41 (39.8)	9 (20.0)	**0.024**
$25,000–49,999	85 (24.0)	50 (24.3)	24 (23.3)	11 (24.4)	0.980
$50,000–74,999	40 (11.3)	29 (14.1)	7 (6.8)	4 (8.9)	0.140
$75,000+	32 (9.0)	13 (6.3)	12 (11.7)	7 (15.6)	0.080
Don’t Know/Refused	61 (17.3)	28 (13.6)	19 (18.4)	14 (31.1)	**0.017**
**Highest Level of Education**					
<High School	57 (16.0)	28 (13.5)	26 (25.2)	3 (6.5)	**0.006**
High School Graduate	111 (31.1)	70 (33.6)	32 (31.1)	9 (19.6)	0.175
Some College	80 (22.4)	59 (28.4)	19 (18.4)	2 (4.3)	**0.001**
College and Beyond	99 (27.7)	43 (20.7)	25 (24.3)	31 (67.4)	**<0.001**
Don’t Know/Not Sure	10 (2.8)	8 (3.8)	1 (1.0)	1 (2.2)	0.419
**Healthcare Coverage**					**<0.001**
Yes	305 (85.4)	193 (92.8)	83 (80.6)	29 (63.0)	
No	52 (14.6)	15 (7.2)	20 (19.4)	17 (37.0)	
**Primary Care Doctor**					**<0.001**
Yes	324 (91.0)	199 (95.7)	89 (87.3)	36 (78.3)	
No	32 (9.0)	9 (4.3)	13 (12.7)	10 (21.7)	
**Routine Physical**					0.154
Within the last year	321 (90.4)	189 (91.7)	94 (91.3)	38 (82.6)	
>1 Year	34 (9.6)	17 (8.3)	9 (8.7)	8 (17.4)	
	**Mean ± SE** **(Range)**	**Mean ± SE** **(Range)**	**Mean ± SE** **(Range)**	**Years ± SE** **(Range)**	**p-value**
**Time in US (years)**					
	44.1 ± 1.11 (0.08–75)	59.7 ± 0.48 (50–75)	24.5 ± 1.30 (0.08–55)	16.7 ± 1.63 (2–43)	**<0.001**

*May not sum to 357 due to missing data. Boldface indicates statistical significance (p < 0.05). SE, standard error.

Adjusted CRCS prevalence and adherence to national CRCS guidelines are presented in **Table 2**. For ever having any type of CRCS, Caribbean immigrants (95.0%) and African immigrants (90.2%) had a higher prevalence when compared to US-born Blacks (59.2%) (p-value <0.001). While the entire study population had a low proportion of stool test adherence (12.9%), US-born Blacks had lower proportions of colonoscopy adherence (40.1%; p-value <0.001) and overall CRCS adherence (45.8%; p-value <0.001), when compared to Caribbean immigrants (80.3% colonoscopy, 51.4% overall CRCS) and African immigrants (82.1% colonoscopy, 80.6% overall CRCS).

**Table 2:** Adjusted prevalence and adherence of colorectal cancer screening for the CAP3 study population (N = 357).

	Overall Sample	US-Born	Caribbean-Born	African-Born	p-value
	N = 357%	N = 208%	N = 103%	N = 46%	
	(95% CI)	(95% CI)	(95% CI)	(95% CI)	
PREVALENCE^a^					
Stool Test^c^	34.6	30.7	42.7	38.5	0.081
	(29.2–40.6)	(21.0–42.5)	(25.9–61.4)	(17.4–65.1)	
Colonoscopy^d^	65.2	41.7	90.9	84.7	**<0.001**
	(58.5–71.3)	(28.9–55.6)	(79.1–96.4)	(61.2–95.1)	
Any CRCS^e^	78.2	59.2	95.0	90.2	**<0.001**
	(72.4–83.1)	(44.4–72.4)	(86.0–98.3)	(71.4–97.1)	
ADHERENCE^b^					
Stool Test^f^	12.9	11.9	17.3	9.3	**0.0430**
	(9.0–18.1)	(6.0–22.3)	(6.5–38.7)	(1.6–38.7)	
Colonoscopy^g^	55.6	40.1	80.3	82.1	**<0.001**
	(48.1–62.8)	(27.9–54.7)	(57.1–92.6)	(54.3–94.6)	
Overall CRCS^h^	52.6	45.8	51.4	80.6	**<0.001**
	(46.6–58.5)	(33.6–58.5)	(35.8–66.9)	(60.0–92.1)	

CAP3, Cancer Prevention Project of Philadelphia; CI, Confidence Interval; CRCS, Colorectal Cancer Screening. Boldface indicates statistical significance (p <0.05).

a Respondent ever had a stool based test in their lifetime; respondent ever had a colonoscopy in their lifetime; respondent ever had a stool based test, colonoscopy or flexible sigmoidoscopy in their lifetime.

b Respondent had a stool based test within the last year; respondent had a colonoscopy within the last 10 years; respondent had a stool test within the last year, a sigmoidoscopy in the last 5 years, or a colonoscopy within the last 10 years.

c Adjusted for length of time in the US, marital status, healthcare coverage, if the participant had a PCP, and income.

d Adjusted for sex, length of time in the US, marital status, healthcare coverage, if the participant had a PCP, whether the participant had a routine physical in the last year, income, and education.

e Adjusted for length of time in the US, marital status, healthcare coverage, if the participant had a PCP, and income.

f Adjusted for sex, length of time in the US, marital status, healthcare coverage, if the participant had a PCP, income and education.

g Adjusted for length of time in the US, marital status, healthcare coverage, if the participant had a PCP, whether the participant had a routine physical in the last year, income and education.

h Adjusted for length of time in the US, marital status, healthcare coverage, if the participant had a PCP, whether the participant had a routine physical in the last year, and education.

Odds ratios for each adjusted model are presented in **Table 3**. The adjusted model for overall CRCS adherence, region of birth, our independent variable of interest, revealed that African immigrants were five times more likely to be adherent when compared to US-born Blacks (OR 5.25; 95% CI 1.34–20.6). Also, in the overall CRCS adherence model, individuals that did not have healthcare coverage were less likely to be adherent (OR 0.24; 95% CI 0.11–0.56). Length of time in the US was also associated with increased odds of overall CRCS adherence, where there are a 4% increased odds of overall CRCS adherence for each year spent in the US (95% CI 1.02–1.07). In the adjusted model for adherence to stool test, no variables of interest were revealed to be statistically significantly associated with adherence. However, it must be noted that length time in the US showed a marginal association (OR 1.01; 95% CI 0.98–1.05). The adjusted model for colonoscopy as a modality of CRCS revealed that Caribbean immigrants had a 6.84 increased odds (95% CI 1.49–231.5) and African immigrants had a 7.14 (95% CI 1.27–40.3) increased odds of adherence when compared to US-born Blacks. Not having healthcare coverage was associated with being 87% less likely to be adherent to colonoscopy (OR 0.13; 95% CI 0.04–0.43). Lastly, length of time in the US was associated with increased levels of adherence in this model as well. Specifically, for each year spent in the US, participants were 6% more likely to adhere to colonoscopy (OR 1.06; 95% CI 1.03–1.10).

**Table 3:** Adjusted logistic regression for the association between region of birth and colorectal cancer screening adherence (N = 357).

	Overall CRCS Adherence^a,d^ N = 350 OR (95% CI)	Stool Test Adherence^b,e^ N = 333 OR (95% CI)	Colonoscopy Adherence^c,f^ N = 235 OR (95% CI)
**Region of Birth**			
US	Ref	Ref	Ref
Caribbean	1.43 (0.52–3.93)	1.61 (0.43–5.96)	**6.84 (1.49–31.5)**
Africa	**5.25 (1.34–20.6)**	1.20 (0.18–7.80)	**7.15 (1.27–40.3)**
**Sex**			
Male	Ref	Ref	Ref
Female	0.76 (0.46–1.27)	0.67 (0.35–1.31)	1.56 (0.82–2.95)
**Length of Time in the US**			
	**1.04 (1.02–1.07)**	1.02 (0.98–1.05)	**1.06 (1.02–1.10)**
**Marital Status**			
Married/Member of Unmarried Couple	Ref		Ref
Divorced, Widowed or Separated	0.75 (0.45–1.25)		0.68 (0.35–1.34)
**Healthcare Coverage**			
Yes	Ref	Ref	Ref
No	**0.24 (0.11–0.56)**	0.67 (0.18–2.47)	**0.13 (0.04–0.43)**
**Primary Care Provider**			
Yes	Ref	Ref	Ref
No	0.68 (0.41–1.12)	0.55 (0.19–1.56)	0.92 (0.46–1.81)
**Routine Physical**			
Within the last year		Ref	
>1 Year		0.27 (0.03–2.18)	
**Annual Household Income**			
≥$50,000			Ref
<$50,000			1.57 (0.72–3.41)
**Highest Level of Education**			
≤High School		Ref	Ref
>High School		0.56 (0.29–1.07)	0.97 (0.51–1.86)

CRCS, Colorectal Cancer Screening; OR, Odds Ratio; CI, Confidence Interval. Boldface indicates statistical significance (p <0.05).

a Adjusted for sex, length of time in the US, marital status, healthcare coverage and if the participant had a PCP.

b Adjusted for sex, length of time in the US, education, healthcare coverage, if the participant had a PCP, and whether the participant had a routine physical in the last year.

c Adjusted for sex, length of time in the US, marital status, income, education, healthcare coverage and if the participant had a PCP.

d Respondent had a stool based test within the last year.

e Respondent had a colonoscopy within the last 10 years.

f Respondent had a stool test within the last year, a sigmoidoscopy in the last 5 years, or a colonoscopy within the last 10 years.

## Discussion

To our knowledge, this is the first study to assess within race differences of overall CRCS and modality-specific CRCS, providing a unique perspective on screening patterns for the Black, heterogeneous racial group. We found that the prevalence of overall CRCS was high in this study population, however, adherence was not ideal. In addition, we found that when we disaggregated the Black population, Caribbean and African Immigrant Blacks had higher proportions of ever having colonoscopy and overall CRCS when compared to US-born Blacks. Further, immigrant Blacks had higher odds of being adherent to colonoscopy recommendations than US-born Blacks.

The overall adjusted CRCS prevalence for this study population was 78.2%, which is higher than the Healthy People 2020 benchmark of 70.5% (40) and prevalence of ever having CRCS reported from other national surveys (40, 41). Specifically, BRFSS and the National Health Interview Survey data from 2013–2018 report CRCS prevalence between 59.1 and 67.8% among Blacks (40, 41). However, data within these national surveys are not reported as granularly as our study; therefore, we are unable to compare prevalence by ethnic sub-groups. Overall, prevalence of stool test within this population was lower than colonoscopy, which is similar to published literature documenting colonoscopy is the most common screening modality, 74.9–84.2%, when compared to stool tests, 5.3–7.5%, from 2012, which is a similar time frame to the current study (41). Further, in a racially diverse population, Hawley et al. reported 37% of participants preferred colonoscopy, while 31% preferred a stool based test (42). Similarly, Palmer et al. found that 57% of individuals who self-identified as Black preferred colonoscopy over stool based testing (43). Our findings show US-born Blacks had lower proportions of both colonoscopy and stool based tests when compared to African and Caribbean immigrants (see **Table 2**). These data suggest a need for targeted intervention towards US-born Blacks to increase CRCS uptake overall, and interventions to increase stool based testing within the Black population as a whole.

The proportions of the study population who were adherent to modality-specific tests and overall CRCS were quite low overall and in comparison to national data (44). For example, 2018 BRFSS reported that among Black respondents, 69.7% met USPSTF recommendations for testing (44), versus ~53% overall adherence in this study. In addition, stool test had the lowest adherence in our study population across all sub-groups (9.3–17.3%), which is similar to Daskalakis et al., Shavers et al., and James et al. where adherence proportions ranged from 8.5–17% study in Black CRCS studies and considerably lower than O’Malley et al. and Waghray et al. that reported adherence proportions at 29–30.9% (25, 45–48). This finding is not surprising in that stool test is recommended in clinical practice less often than colonoscopy (49–56). However, contrary to our hypothesis, Caribbean immigrants had significantly higher stool test adherence compared to US-born respondents and both Caribbean and African immigrants were significantly more likely to be up-to-date with colonoscopy compared to US-born respondents (80.3–82.1% vs. 40.1%). While seeing a primary care provider facilitates the process/initiation of CRCS and may increase CRCS uptake (57–59), this does not explain the differences we observed. Specifically, there were no differences in having a routine physical in the last year by ethnic sub-group. Further, a higher proportion of US-born Blacks reported having a primary care doctor and health insurance (see **Table 1**). While these are the data, it could be that there was differential over reporting of CRCS in our sample. While the published literature shows that self-report and medical record data for cancer screening measures generally coincide, ethnic and racial minorities tend to over-report screening more than their white counterparts (60–66). While these data offer insights for the aggregate Black population, ethnic sub-group data are not available; thus, it is unclear whether immigrant Blacks over-reported CRCS compared to US-born Blacks. Lofters et al. assessed self-reported validity in a diverse Canadian population and found that all immigrants were more likely to over-report when compared to Canadian naturals (67). Still, this data did not disaggregate the immigrant population to make a clear distinction as to what ethnic groups or countries compromised this group. Further work to examine the agreement of self-reported and actual CRCS within the Black population is warranted to determine the validity of our findings.

Adjusted logistic regression analyses revealed that African immigrants have a 7-fold increased odds of overall CRCS adherence compared to US-born Blacks and both Caribbean and African immigrants were more likely to be adherent to colonoscopy. In addition, not having health insurance was independently associated with reduced odds of adherence to overall CRCS and colonoscopy. Surprisingly, there was no association between having a regular PCP and overall CRCS adherence (**Table 3**). Higher adherence among immigrant Blacks was contradictory to our original hypothesis. This could be influenced by length of time in the US, which was independently associated with increased odds of overall CRCS and colonoscopy adherence, as well as medical mistrust. For example, the availability of screening programs in the immigrant home country may be non-existent, which is the case for a majority of Caribbean and African countries (68, 69). Thus, immigrants may take advantage of preventive screening that has been previously inaccessible to them. Relatedly, medical mistrust and/or distrust of the US health infrastructure among US-born Blacks (70–73) may explain why they are less likely to be adherent to overall CRCS and colonoscopy when compared to immigrant Blacks. A long history of mistreatment of US-born Blacks in medicine and health related research is documented most famously by the Tuskegee study of “Untreated Syphilis in the male Negro”, and has left a lasting, negative impact on US-born Blacks (70, 74–76). Events of the past are further exacerbated by the current social climate (77) and the disproportionate rates in which diseases affect the Black community. This mistrust of the healthcare system may be more innate in US-born Blacks when compared to immigrant Blacks, because these types of social injustices are not as common in their home countries. Subsequently, immigrant Blacks may be more likely to place their trust in healthcare professionals than their US-counterparts (78).

Finally, while our adjusted analyses revealed a higher odds of adherence to overall CRCS and colonoscopy among immigrant Blacks when compared to US-born Blacks, crude analyses for CRCS (data not shown) showed that lower proportions of immigrant Blacks, were up-to-date on screening when compared to their US-born counterparts. Thus, CRCS interventions to increase coverage and utilization of healthcare are warranted to ensure CRCS uptake in the heterogeneous Black population.

This study provides novel CRCS findings within the heterogeneous Black population, which is a major strength of this work. For the first time, we report within race differences (i.e. US-born, Caribbean and African Immigrant Blacks) for overall and modality-specific CRCS prevalence and adherence; and examined the association of region birth with overall and modality-specific CRCS adherence. There is scant literature on CRC screening in immigrant populations of those identifying as Black; thus, this study provides insight and begins to address a gap in the literature. Immigrant health is an emerging topic in the literature and this paper provides novel information within this body of research. In addition to our unique study population, our survey instrument allowed us to code prevalence and adherence similarly to other national surveys (30, 31, 79) making the overall sample data for Blacks comparable to national reports.

While this paper provides insights on CRCS within a heterogeneous Black population, there are limitations. This study, like other survey-based studies, is subject to various types of biases. First, study participants had to recall the type of CRCS as well as the length of time since their last CRCS, which could bias our findings. For example, telescoping may have occurred, where respondents were likely to report their CRCS to be more recent than it actually was. Recall bias could also have contributed to misclassification of screening type, where respondents recalled the wrong screening type or the wrong date since their last CRCS. However, previous work has shown that the two to three part nature of the CRCS questions used, which were identical to those in a validated, national based survey (31), reduced the likelihood of telescoping (80). Also, recall bias could have contributed to missing data on CRCS use (data not shown); however, it is highly unlikely that our findings were affected by these missing data because only about 1.1% (n = 4) of all CRCS data were missing. In addition, this study included volunteer participants; thus there are no non-responders for comparison. Further, the validity of self-reported CRCS is quite high compared to medical records (61–65, 81), which limits bias. Second, this survey was interviewer-administered, which may have introduced social desirability bias where respondents felt that they had to provide the interviewer with socially acceptable answers indicating they had screening in the appropriate timeframe. This type of bias would have subsequently led to non-differential misclassification bias (39), which likely would not have a significant impact on our findings. Third, our length of time in the US variable assumed that all US-born Blacks lived in the US their entire lives. Although the data on US expatriates are limited (82–84), approximately 9 million US-born individuals live abroad for 5–10 years. To explore whether this could have impacted our findings, we conducted a post-hoc sensitivity analysis based on a liberal assumption that 10% of US-born Blacks lived outside of the US for 5 years. Findings from this analysis assessing the association of region of birth with overall CRCS and colonoscopy were almost identical to the original analyses (data not shown). Fourth, cross-sectional studies generally have inherent limitations given unknown temporality; however, it was not an issue for these analyses, as region of birth preceded CRCS. Fifth, obtaining CRCS can be difficult (85–90); however, CRCS barriers, which include among other things, fear, logistics of the test, lack of information, time, and lack of physician recommendation were not assessed, which could impact our findings. Had we been able to incorporate CRCS barriers in our regression models, the odds ratios could have been attenuated towards the null. Limited generalizability, is also a limitation of this study. Participants in this study were a specific sample of persons who self-identified as Black of Philadelphia and as such are not necessarily representative of the CRC screening population in the US. This, data may only be comparable to cities that are also majority Black and have similar proportions of immigrant Blacks from Africa and the Caribbean. Aligned with this, while the region of birth variable included multiple countries across the Caribbean and Africa, it must be noted that the majority of Caribbean immigrants came from Haiti (69.9%), followed by Jamaica (19.4); and African immigrants came from Nigeria (67.4%) and Liberia (15.2%) (data not shown). Subsequently, the generalizability of this data to all immigrants from these regions is limited. Also, while we powered to observe significant differences between region of birth and overall CRCS and colonoscopy we were drastically underpowered to observe such differences for stool based CRCS. In order to observe a statistically significant difference between region of birth and stool tests, we would have need over 1,100 participants at 80% power, with a two-tailed test with α = 0.05. Lastly, we did not differentiate between screening and diagnostic colonoscopy after stool-based CRCS. However, given the very low prevalence of stool test in our study population, it is likely that the majority of colonoscopies were for screening purposes and not diagnostic, subsequently having no meaningful effect on our findings.

In summary, self-reported overall adherence to CRCS and modality specific CRCS are sub-optimal among self-identified Blacks in Philadelphia. While immigrant Blacks were more likely to be adherent to colonoscopy when compared to US-Born Blacks, CRCS was still sub-optimal across all ethnic sub-groups, suggesting that interventions to increase adherence should be targeted to the entire US-Black population. This study provides the first data on CRCS and region of birth among a heterogeneous Black population that has historically been underrepresented in research. To advance CRCS research particularly in immigrant and traditionally underserved populations, future studies could assess CRCS in the expanded CAP3 population, which now includes populations in California and the Caribbean. In addition, future studies should explore CRCS barriers to better understand what might be influencing CRCS in heterogeneous Black populations and whether these barriers are nuanced by culturally specific factors.

## Data Availability Statement

The raw data supporting the conclusions of this article will be made available by the authors, upon reasonable request.

## Ethics Statement

The studies involving human participants were reviewed and approved by Fox Chase Cancer Center Institutional Review Board. The patients/participants provided their written informed consent to participate in this study.

## Author Contributions

EB and CR had full access to all the data and take responsibility for the integrity of the data and the accuracy of the data analysis study conception and design. CR is responsible for the original cohort study design. The subset analysis of CAP3 data was designed and conceptualized by EB. Statistical analysis and drafting of manuscript was done by EB. Critical revision of the manuscript for intellectual content was done by RJ. CR is responsible for study supervision, administrative, technical, and material support. All authors contributed to the article and approved the submitted version.

## Funding

The project was originally funded by institutional funds provided by Fox Chase Cancer Center (CCSG 5P30CA006927) and a Research Scholar Grant from the American Cancer Society (PI Ragin: RSG-14-033-01-CPPB) and the NCI (PI Ragin/Fang: 5U54CA221705).

## Conflict of Interest

The authors declare that the research was conducted in the absence of any commercial or financial relationships that could be construed as a potential conflict of interest.

## Publisher’s Note

All claims expressed in this article are solely those of the authors and do not necessarily represent those of their affiliated organizations, or those of the publisher, the editors and the reviewers. Any product that may be evaluated in this article, or claim that may be made by its manufacturer, is not guaranteed or endorsed by the publisher.
